# Corrigendum

**DOI:** 10.1002/jcla.24159

**Published:** 2022-01-07

In Volume 35, Issue 6, 2021, in the title “Screening of tumor grade‐related mRNAs and lncRNAs for Esophagus Squamous Cell Carcinoma” by Xin Gao, Qian Liu, Xue Chen, Shaoping Chen, Jianmei Yang, Qiang Liu, Yufeng Cheng, there was a mistake in Validation of qRT‐PCR in results section as published. “Except for RP11‐25G10.2, the other five genes were consistent with our TCGA analysis.” should be changed to “Except for RP11‐25G10.2 and ATP1A2, the other four genes were not consistent with our TCGA analysis.”

A correction has been also made to the Abstract section under “Results”:

“Except for RP11‐25G10.2, the other five genes were consistent with our TCGA analysis.” should be changed to “qRT‐PCR results showed that only RP11‐25G10.2 and ATP1A2 were consistent with our TCGA analysis.”

The authors apologize for these errors and state that they do not change the scientific conclusions of the article in any way.

